# A role for miR-132 in learned safety

**DOI:** 10.1038/s41598-018-37054-z

**Published:** 2019-01-24

**Authors:** Marianne Ronovsky, Alice Zambon, Ana Cicvaric, Vincent Boehm, Bastian Hoesel, Bernhard A. Moser, Jiaye Yang, Johannes A. Schmid, Wulf E. Haubensak, Francisco J. Monje, Daniela D. Pollak

**Affiliations:** 1 Department of Neurophysiology and Neuropharmacology, Center for Physiology and Pharmacology, Medical University of Vienna, Vienna, Austria; 2 Research Institute of Molecular Pathology, Vienna, Austria; 3 Department of Vascular Biology and Thrombosis Research, Center for Physiology and Pharmacology, Medical University of Vienna, Vienna, Austria

## Abstract

Learned safety is a fear inhibitory mechanism, which regulates fear responses, promotes episodes of safety and generates positive affective states. Despite its potential as experimental model for several psychiatric illnesses, including post-traumatic stress disorder and depression, the molecular mechanisms of learned safety remain poorly understood, We here investigated the molecular mediators of learned safety, focusing on the characterization of miRNA expression in the basolateral amygdala (BLA). Comparing levels of 22 miRNAs in learned safety and learned fear trained mice, six safety-related miRNAs, including three members of the miR-132/-212 family, were identified. A gain-of-function approach based upon *in-vivo* transfection of a specific miRNA mimic, and miR-132/212 knock-out mice as loss-of-function tool were used in order to determine the relevance of miR-132 for learned safety at the behavioral and the neuronal functional levels. Using a designated bioinformatic approach, PTEN and GAT1 were identified as potential novel miR-132 target genes and further experimentally validated. We here firstly provide evidence for a regulation of amygdala miRNA expression in learned safety and propose miR-132 as signature molecule to be considered in future preclinical and translational approaches testing the transdiagnostic relevance of learned safety as intermediate phenotype in fear and stress-related disorders.

## Introduction

As fear can be generated and increased by learning processes, likewise also learned fear inhibitory mechanisms exist. One of these mechanisms is referred to as “learned safety” as it involves the learning about safety signals, which act to inhibit conditioned fear in the summation and retardation tests^1–5^.

Similar to learned fear, the learned safety paradigm can be examined experimentally in humans as well as in animals, including rodents^1,6^. Learned fear is studied by a fear conditioning paradigm in which a neutral conditioned stimulus (CS) such as a tone is positively correlated with an aversive unconditioned stimulus (US) such as an electric shock. In contrast, in the learned safety paradigm, CS and US are always presented unpaired, and therefore the CS is perceived as a signal of safety, indicating protection from imminent danger^1^. Learned safety signals exert effects beyond the regulation of fear responses: through the identification of episodes of security they also relate to positive affective states, elicit a reward-related approach and a reduction of depression-like behavior in mice^2,3,7^.

A candidate structure involved in learned safety processing is the amygdala. It was previously demonstrated that neural activity and spine synapse size are reduced upon learned safety in this particular brain region^3,7,8^. A detailed electrophysiological characterization revealed the presence of specific “safety neurons” in the basal amygdala, which could be further distinguished by either a selective response to safety cues alone or activation in response to safety cues coupled to a rewarding stimulus^9^. This cellular analysis further complemented previously obtained behavioral data^2,3,7^ supporting the notion of shared neural mechanisms of safety and reward processing in the amygdala. While elegantly delineated at the behavioral and neural levels, information on the molecular underpinnings of learned safety is limited. In our previous study, we investigated gene expression changes in the BLA underlying the acquisition of learned safety and reported a specific “fingerprint” of mRNA expression in the BLA^2^. However, how gene expression is regulated in response to the recall of learned safety has so far remained unexplored, which led us to examine the involvement of specific microRNAs (miRNAs) as molecular mediators.

miRNAs are small noncoding RNAs regulating gene expression on the posttranscriptional level and have been demonstrated with a multitude of functions in the physiology and pathology of the brain^10–13^. In several studies, a role for miRNAs in amygdala-dependent fear learning was already demonstrated^14,15^. Furthermore, miRNAs in the BLA exert crucial functions in fear extinction, a learned safety-related fear inhibitory paradigm, proposing an involvement of miRNAs in inhibitory emotional learning and memory^16,17^.

Using a focused, discovery-driven approach led us to the detection of six safety-associated miRNAs, three of which belong to the cluster of miRNAs forming the miR-212/-132 family, whose members are highly relevant for the neuronal function during development and in adulthood and have been implicated in several disorders of the brain (see for review^18^). We therefore here decided to further characterize the role of miR-132 in learned safety behavior *in-vivo* and in BLA functional activity *ex-vivo* and identify novel miR-132 target genes using combined *in-silico* and *in-vitro* approaches.

## Materials and Methods

### Animals

8–12 weeks old male C57Bl/6N mice were used for all wildtype experiments. miR-132/212 knockout mice have been previously described^19^. miR-132/212 knockout mice were generated by crossing heterozygous parents and compared to wildtype littermate controls for all experiments. All animals were housed under standard conditions and experiments followed the ARRIVE guidelines and the U.K. Animals Scientific Procedures Act, 1986 and associated guidelines (EU Directive 2010/63/EU for animal experiments) and were approved by the national ethical committee on animal care and use (BMWF-66.009/0200-WF/V/3b/2016; Bundesministerium für Wissenschaft und Forschung).

### Conditioning

Conditioning procedures followed a previously published protocol^2^. All mice were handled once per day for two days prior to experiments, in order to accustom the animals to the experimenter. At least 30 minutes before the conditioning trial, mice were transported to a habituation room close to the experimental room. In all instances the unconditioned stimulus (US) consisted of a 0.6 mA electric shock and the conditioned stimulus (CS) of a white noise at 75 dB. Following the end of the conditioning session, mice were removed from the conditioning chamber, placed back into their home cage and returned to the habituation room. The chamber was carefully cleaned with 70% ethanol after each animal.

**Figure 1:**
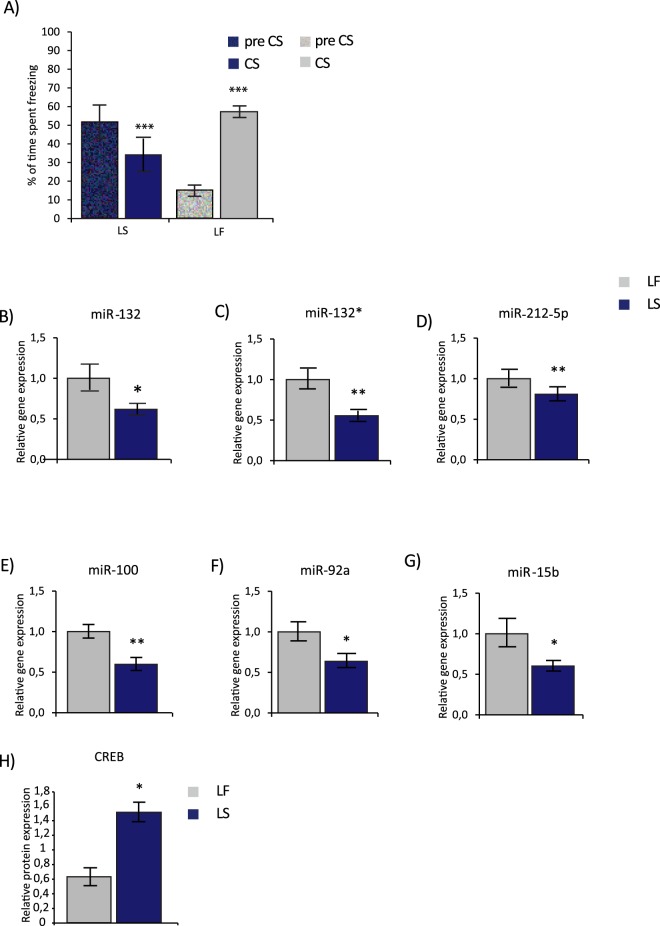
BLA miRNA expression in learned safety (LS) and learned fear (LF). (**A**) Percentage of time spent freezing before (preCS) and during (CS) presentation of the conditioned stimulus in LS and LF trained mice (n = 13–14 per group). Relative miRNA expression of (**B**) miR-132, (**C**) miR-132*, (**D**) miR-212-5p, (**E**) miR-100, (**F**) miR-92a and (**G**) miR-15b determined by qRT-PCR in BLA tissue of LS and LF mice sacrificed 2 hours after the memory test. Results were normalized to SNORD61 as reference RNA and plotted relative to the mean of LF (n = 7-8 per group). (**H**) Relative CREB protein expression in BLA tissue of LS and LF mice sacrificed 12 hours after the memory test revealed by Western Blot (n = 3 per group). Data are displayed as mean ± SEM. *p < 0.05, **p < 0.01, ***p < 0.001.

**Figure 2:**
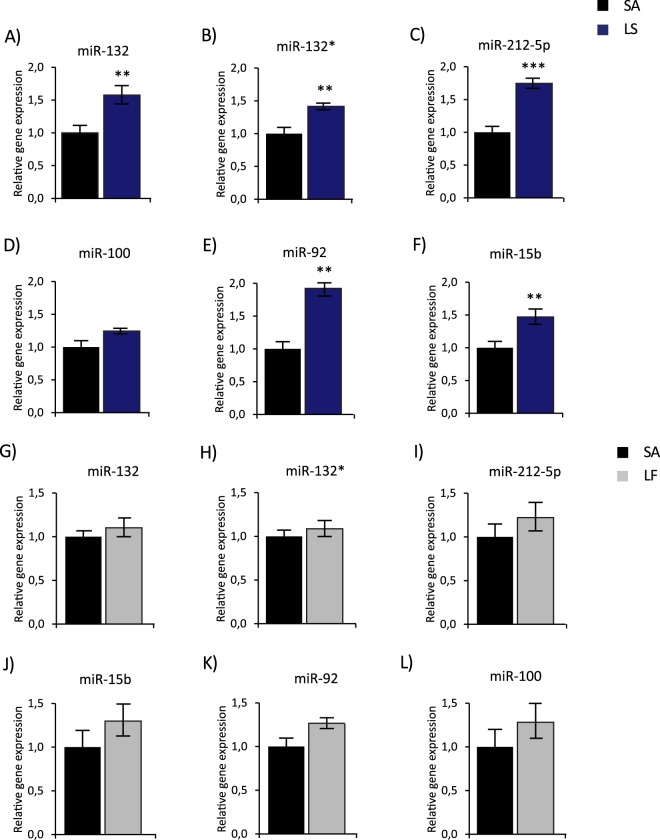
BLA miRNA expression in learned safety (LS), learned fear (LF) and shock alone (SA) controls. Relative miRNA expression of (**A**) miR-132, (**B**) miR-132*, (**C**) miR-212-5p, (**D**) miR-100, (**E**) miR-92a and (**F**) miR-15b determined by qRT-PCR in BLA tissue of LS mice compared to SA mice 2 hours after the memory test. Results were normalized to SNORD61 as reference RNA and plotted relative to the mean of the SA group (n = 6–7 per group). Relative miRNA expression of (**G**) miR-132, (**H**) miR-132*, (**I**) miR-212-5p, (**J**) miR-100, (**K**) miR-92a and (**L**) miR-15b determined by qRT-PCR in BLA tissue of LF mice compared to SA mice 2 hours after the memory test. Results were normalized to SNORD61 as reference RNA and plotted relative to the mean of the SA group (n = 7–8 per group). All data are displayed as mean ± SEM. *p < 0.05, **p < 0.01, ***p < 0.001.

For the learned safety and the matching learned fear procedure (Figs 1 and 2A–F) mice were trained on three consecutive days with one training session per day lasting a total 680 seconds. Each training session comprised four CS and four US presentations, which were delivered in an explicitly unpaired manner for learned safety and in a paired presentation mode for learned fear. For the “traditional” fear conditioning protocol, mice received one training session per day (two paired CS-US presentations), lasting for 360 seconds on two consecutive days. Shock-alone controls were exposed to the US only with identical times spent in the conditioning chamber as the learned safety and learned fear groups.

In each instance, a memory recall test was performed 24 hours after the last training session which lasted for 210 seconds and comprised a single CS presentation. All behavioral experiments were recorded using the near-infrared (NIR) Video Conditioning System from Med Associates (MedAssociates Inc., St. Albans, USA) and analyzed by the Video Freeze® (MedAssociates Inc.) software.

### Stereotactic Surgery

The stereotactic procedure was conducted following a previously published procedure^20^. Briefly, guide cannulas (Plastics One, Roanoke, USA; cut length: 5 mm, 24 gauges) were introduced 1 mm above the target site (BLA) at AP: −1.4 mm, ML: +/−3.15 mm, DV: −5 mm. All animals were allowed a 2 weeks recovery period before further experiments.

### *In-vivo* miRNA transfection

For *in-vivo* transfection of a miR-132 mimic, the jetSI™ 10 mM *in-vivo* transfection reagent (Polyplus-transfection SA, Illkirch, France) was prepared according to the manufacturer’s instructions. 0.5 µL of the miRNA-132 mimic or the negative control (*C*.*elegans* miR-67 mimic) (both: GE Healthcare Dharmacon Inc., Lafayette, USA) were injected into the BLA at a rate of 0.25 µL/min.

### Electrophysiology

Coronal slices (300 µm) containing the BLA were prepared from mice 24 hours after *in-vivo* transfection for gain-of-function experiments and from miR-132 KO and WT animals for loss-of-function experiments. Long-term potentiation (LTP) was induced and recorded according to published methods^21^. In brief, animals were anesthetized via i.p. injection of a ketamine/xylazine cocktail at 100 mg/kg ketamine (Ketanest® S, Pfizer Corporation Austria Gesellschaft m.b.H., Vienna, Austria) and 40 mg/kg xylazine (Rompun®, Bayer, Germany) and transcardially perfused with ~20 mL of a saline cutting solution containing (in mM): 220 Sucrose, 2.4 KCl, 26 NaHCO3, 10 Glucose, 5 L-Ascorbate, 3 Sodium Pyruvate, 0.5 CaCl2, 10 MgCl2. Brains were quickly dissected and sliced in frosty cutting solution under constant carbogen perfusion (95% O2/5% CO2) using a vibratome (Vibrating Microtome 7000 Model 7000smz-2, Campden Instruments Ltd., Loughborough, Leics., U.K.). Slices were then transferred to a home-made recovery chamber containing an artificial cerebrospinal fluid (aCSF) solution containing (in mM): 118 NaCl, 2.5 KCl, 1 NaH2PO4, 26.2 NaHCO3, 20 Glucose, 2 CaCl2, 1 MgCl2, and slices rested during at least 60 min at 33 °C under constant carbogen perfusion. For electrophysiology recordings, each slice was transferred to a recording chamber superfused with carbogen-aCSF solution and electrically stimulated via a custom-built bipolar tungsten electrode insulated to the tip and located at the lateral nucleus of the amygdala, right below the cortical inputs endings. For recordings, pipettes from borosilicate glass capillaries were prepared using a horizontal puller (Sutter Instrument, Novato, CA, USA) backfilled with aCSF (resistance 2–4 MΩ) and placed at the upper portion of the basolateral nucleus^22^. Stimulation was delivered using an ISO-STIM 01D isolator stimulator (NPI Electronics, Tamm, Germany). Input/output curves were obtained from the changing values in field decaying slopes upon delivery of incremental pulses of voltage (0–7 V, 1 V increments, 200 μs duration) and used to determine the strength of synaptic transmission. For synaptic potentiation recordings, stable basal synaptic transmission baselines were recorded for at least 10 min using a stimulus input (200 μs duration, 0.03 Hz) eliciting ~30% of maximal input/output value. LTP was induced by delivering 2 trains of only 0.5 s of high frequency electrical stimulation (100 Hz, 200 μs/pulse, 10 s intertrain interval) also applying the stimulus amplitudes that elicited ~30% of maximal synaptic response as determined from input/output curves (intensity values which elicited comparable field responses in all experimental groups). Elicited fEPSPs were subsequently recorded for at least 30 min. Synaptic strengthening was determined by examining the temporal progression of the linear slope decays of fEPSPs induced after high frequency stimulation normalized to respective baseline values. All recordings were conducted using an AxoClamp-2B amplifier (Bridge mode) and a Digidata 1550A interface (Axon Instruments, Molecular Devices, Berkshire, UK). Data analysis was performed using the pClamp10 Software (Molecular Devices, Sunnyvale, CA, USA).

### Brain dissection and micropunch procedure

Mice were sacrificed via neck dislocation, brains were rapidly dissected over ice and stored in optimal cutting temperature compound at −80 °C for collection of BLA samples using a micropunch procedure^23^ and manual dissection of hippocampal tissue.

### Cell culture and *in-vitro* transfections

3t3 cells (American Type Culture Collection (ATCC), Manassas, USA) were maintained in DMEM medium (Gibco, Thermo Fisher Scientific Inc., Waltham, USA) containing 10% fetal bovine serum (Sigma-Aldrich, Vienna, Austria), PenStrep (100 U/mL Penicilium and 100 μg/mL Streptomycin) (Gibco, Thermo Fisher Scientific Inc.) and Glutamine (2 mM) (Gibco, Thermo Fisher Scientific Inc.) and transfected with 0.1 nM miR-132 mimic or miRNA mimic negative control (both from GE Healthcare Dharmacon Inc.) diluted in lipofectamine 2000 (Invitrogen, Carlsbad, USA). Cells were harvested 24 hours after the transfection.

### RNA extraction, cDNA synthesis and qRT-PCR

#### RNA extraction and cDNA synthesis

Total RNA was extracted using a commercial kit (miRNeasy microKit, Qiagen, Hilden, Germany). cDNA synthesis from mature miRNAs was obtained using the miScript II RT Kit (Qiagen). For cDNA synthesis from mRNA the DyNamo Kit (Thermo Fisher Scientific Inc.) was used. 500 ng of RNA was used for cDNA synthesis in each case. Manufacturer’s instructions were followed in all instances.

#### qRT-PCR

For miRNA qRT-PCR the miScript SYBR® Green PCR Kit (Qiagen) and for mRNA the SYBR® Green PCR Master Mix (Applied Biosystems®) was used. In both instances, the manufacturer’s instructions were followed. Each cDNA sample was tested in duplicate (as in comparable studies^24–26^ and dCT was determined by normalization of the Ct value to the Ct value of the reference small nucleolar RNA SNORD61 (Qiagen) for miRNA or to beta-actin (Invitrogen, Carlsbad, USA) for mRNA. Data were transformed into relative values by calculating: 2−ΔΔCT (ddCT) as previously described^23^.

### Protein isolation and Western Blot

Proteins were purified from 3t3 cells and BLA tissue. Cells and tissue were lysed using a lysis buffer containing Tris pH 7.4 50 mM, NaCl 150 mM, EDTA 5 mM, Triton X-100 1%, protease inhibitors cocktail (Roche, Mannheim, Germany) and protease and phosphatase inhibitors cocktail (ThermoScientific, Rockford, USA)). Cell/tissue homogenate was sonicated and total protein content was quantified using a commercial kit (Pierce^TM^ BCA Protein Assay Kit, Thermoscientific) with assistance of the software GEN5 (BioTek, Vermont, USA).

Western Blot analysis was conducted using the following primary antibodies and dilutions: anti-PTEN: 1:1000, ab154812 (Abcam, Cambridge, UK), anti-beta actin 1:2000, A0760–40 (USBiological, Massachusetts, USA) and anti-GAPDH 1:3000, ab9485 (Abcam), anti-CREB 1:250, 48H2 (Cell Signalling, Boston, Massachussets). Secondary antibody signals were detected using the Clarity^TM^ Western ECL detection kit (BioRad, California, USA) and the software Fluorchem^TM^ HD2 (Alpha Innotech, Biozym, Vienna, Austria). Quantification of optical densities was performed using the program ImageJ (Maryland, USA) and values were normalized to those of housekeeping genes (beta actin and GAPDH).

### Bioinformatics

To predict potential target genes for miR-132, the platforms targetscan (http://www.targetscan.org), Miranda (http://www.microrna.org/microrna/home.do) and EIMMo3 (https://www.mirz.unibas.ch/) were used as miRNA target prediction tools. Predicted target genes were further evaluated using the online tool AmiGO (http://amigo.geneontology.org/amigo), the Vienna RNA webserver (RNAfold, http://rna.tbi.univie.ac.at/cgi-bin/RNAWebSuite/RNAfold.cgi) and the Allen mouse brain atlas (http://mouse.brain-map.org/).

### Luciferase Assay

GAT1, PTEN and MeCP2 3´UTR fragments were amplified from genomic DNA using a Q5 High-Fidelity Taq Polymerase, digested with NheI and NotI and cloned in the pmirGLO Dual-Luciferase reporter vector (Promega GmbH, Mannheim, Germany)). All plasmids were sequenced before use. The following primers were used for cloning of GAT1, PTEN and MeCP2 fragments containing a predicted miR-132 binding site: GAT1_fwd: atattagctagccgaccaccacttgatgtctg and GAT1_rev: atattagcggccgcaaaatgcccttttcctgtg; PTEN_fwd: atattagctagctgtgtaatcaaggccagtgc and PTEN_rev: atattagcggccgctcttttttttgtgtgcag; MeCP2_fwd: atattagctagcaaatcgacgcccgagttag and MeCP2_rev: atattagcggccgcgaaaattcctttcacccacca. Plasmids were co-transfected with the miR-132 mimic or a negative control (*C*.*elegans* miR-67 mimic) into Hela cells using DharmaFECT Duo transfection reagent (GE Healthcare Dharmacon Inc., Lafayette, CO, USA) according to the manufacturer’s protocol. Firefly luciferase activity was assessed as previously described^27^ using the Renilla-Glo Luciferase System (Promega) according to the manufacturer’s protocol.

### Experimental Design and Statistical Analysis

The Grubb’s test was used to determine significant outliers. For statistical comparisons of two experimental groups an unpaired two-tailed Student’s *t* test was conducted. Statistical analysis of three or more experimental groups was performed by an analysis of variance (ANOVA). Within- and between-subject variables were evaluated in a mixed model design ANOVA, with the repeated measure as within- subject variable and the injection/genotype (miR-132 mimic or *C*. *elegans* control; miR-132 KO or WT) as the between-subject variable. All statistical analyses were performed using the software SPSS (IBM, Rochester, USA) and p ≤ 0.05 was used as a criterion to define statistical significance in all instances.

## Results

### BLA miRNA expression in learned safety

We used a hypothesis-driven screening approach in order to examine the expression of specific miRNAs in the BLA of learned safety trained mice. Candidate miRNAs had been selected by a literature search focusing on functional categories considered of relevance for learned safety using the search term “miRNA” in combination with one of the following key words: “amygdala; stress; depression; anxiety; learning and memory”. This screening led to the nomination of 22 candidate miRNAs (Table 1) whose expression in response to learned safety was surveyed in the mouse BLA, probing both sense and anti-sense forms. To this end, adult male C57Bl/6N mice were randomly assigned to two groups and trained and tested for either learned safety or learned fear using an established procedure based upon a three days protocol of explicitly unpaired/paired CS-US training^2^. The direct comparison to a learned fear group trained in a paradigm matching in numbers the CS and US presentations used for the learned safety protocol was selected as relevant and most suitable control group accounting for effects induced both by the experience of the CS and US, as also used in our previous study^2^. The memory recall test provided evidence for the expected significant reduction in the percentage of time spent freezing in response to the CS in learned safety trained and a significant increase in learned fear trained mice (F(1, 25) = 92.45, p = 0.0001, mixed-model repeated-measure ANOVA: time x training (LS/LF); Fig. 1A)). Quantitative real-time PCR (qRT-PCR; samples assayed in technical duplicates, mean SEM of duplicates = 0.1 cT) revealed that the expression of miR-132, miR-132*, miR-212-5p, miR-100, miR-92a and miR-15b was significantly reduced in the BLA of learned safety mice (miR-132 t(13) = 2.375, p = 0.034; miR-132* t(12) = 3.447, p = 0.005; miR-212-5p t(13) = 3.354, p = 0.005; miR-100 t(13) = 3.359, p = 0.005; miR-92a t(13) = 2.65, p = 0.02; miR-15b t(13) = 2.388, p = 0.033; unpaired two-tailed Student’s t test) 2 hours after the memory test; Fig. 1B–G). No significant group differences for the remaining miRNAs were observed (Table 2). The activity-dependent transcription factor CREB has been described to regulate four out of the five exclusively safety-related miRNAs (i.e. miR-132, miR-132*, miR-212-5p and miR-15b). We therefore set-out to examine BLA CREB expression in learned safety trained mice and found significantly higher CREB protein levels in BLA tissue of learned safety as compared to learned fear trained animals (CREB: t(4) = 2.794, p = 0.049; unpaired two-tailed Student’s t test (Fig. 1H)) 12 hours after the memory test.

**Table 1:** miRNAs selected for analysis in the BLA of learned safety and learned fear mice.

miRNA	Effect	Reference
miR-15a	expression level changes after acute stress in mouse amygdala	^55^
miR-15b	expression level changes after acute stress in mouse amygdala	^55^
miR-34c	expression level changes after acute stress in mouse amygdala	^55^
miR-34a	expression level changes after acute stress in mouse amygdala chronic treatment with valproate and lithium changes expression level in rat hippocampus	^55, 56^
miR-92a	expression level changes after acute stress in mouse amygdala	^55^
miR-100	expression level changes after acute stress in mouse amygdala	^55^
miR-134	downregulates CREB and BDNF, SIRT1 inhibits expression of miR134 plasma levels of miR134 decreased in manic patients increased expression level after acute stress in rat amygdala	^57– 59^
miR-132	targets BDNF and MeCP2 is regulated by the CREB pathway	^52, 60^
miR-18a	reduces glucocorticoid receptor levels in neuronal cell culture	^61^
miR-183	increased expression level after acute stress in rat amygdala	^59^
miR-212	most highly expressed in hippocampus and amygdala compared to various brain regions	^62^

**Table 2:** Expression of 22 selected miRNAs in the BLA of learned safety (LS) compared to learned fear (LF) mice 2 hours after the memory test.

miRNA	Mean ctrl (LF) ± SEM	Mean LS relative to ctrl (LF) ± SEM	p-value	t-value
miR-15a*	1 ± 0,11	0,69 ± 0,08	0,052	4,658
miR-100*	1 ± 0,16	0,75 ± 0,05	0,178	1,912
miR-183	1 ± 0,18	0,73 ± 0,08	0,217	1,755
miR-34a	1 ± 0,09	0,83 ± 0,09	0,285	1,336
miR-18a	1 ± 0,09	0,90 ± 0,05	0,349	0,821
miR-134*	1 ± 0,08	0,91 ± 0,07	0,465	0,559
miR-34a*	1 ± 0,28	1,21 ± 0,10	0,556	0,239
miR-134	1 ± 0,11	1,09 ± 0,07	0,565	0,3
miR-34c*	1 ± 0,08	0,85 ± 0,07	0,594	0,304
miR-212-3p	1 ± 0,21	0,87 ± 0,10	0,6	0,273
miR-183*	1 ± 0,11	0,82 ± 0,18	0,655	0,522
miR-18a*	1 ± 0,30	0,81 ± 0,26	0,719	0,137
miR-34c	1 ± 0,17	0,89 ± 0,24	0,761	0,104
miR-15b*	1 ± 0,38	0,64 ± 0,27	0,779	0,356
miR-15a	1 ± 0,15	0,94 ± 0,10	0,782	0,071
miR-92a*	1 ± 0,21	0,89 ± 0,09	0,801	0,098

Relative miRNA expression determined by qRT-PCR in BLA tissue of LS and LF mice 2 hours after the memory test. Results were normalized to SNORD61 as reference RNA and plotted relative to the mean of LF (n = 7-8 per group). Data are displayed as mean ± SEM.

To further characterize the relevance of this change in expression with regards to the fear inhibitory and mood-enhancing properties of the memory recall for learned safety, we compared levels of miRNAs with significant differences between safety and fear also between learned safety and a shock alone group in a separate experiment. The expression of miR-132, miR-132*, miR-212-5p, miR-100, miR-92a and miR-15b was significantly increased in the BLA tissue of the learned safety group (miR-132 t(13) = 3.34, p = 0.005; miR-132* t(13) = 3.54, p = 0.004; miR-212-5p t(12) = 5.412, p = 0.000; miR-100 t(9.241) = 2.349, p = 0.043; miR-92a t(11) = 5.073, p = 0.000; miR-15b t(13) = 3.235, p = 0.007; unpaired two-tailed Student’s t test) 2 hours after the memory test (Fig. 2A–F). Additionally, we employed a “traditional” fear conditioning protocol based upon 2 CS-US pairings to compare BLA miRNA expression between learned fear and shock alone groups. No significant group differences in BLA expression of miR-132, miR-132*, miR-212-5p, miR-100 and miR-15b were observed, while miR-92a levels were significantly higher in learned fear as compared to shock alone. (miR-132 t(10.857) = 0.827, p = 0.426; miR-132* t(11) = 0.681, p = 0.51; miR-212-5p t(8.833) = 1.338, p = 0.214; miR-100 t(11) = 1.059, p = 0.312; miR-92a t(9) = 4.704, p = 0.001; miR-15b t(9.707) = 0.721, p = 0.488; Fig. 2G–L).

We next focused on the members of the miR-212/-132 family in order to investigate the temporal specificity of the observed change in BLA miRNA expression of learned safety mice. No differences in BLA expression of miR-132, miR-132*, miR-212-5p were found 24 hours after the memory test (p > 0.05 for all analyzed miRNAs; unpaired two-tailed Student’s *t* test) (Table 3). Evidence for regional specificity was obtained as no differences in the expressional profile of miR-132, miR-132* and miR-212-5p in hippocampal tissue of safety and fear trained mice was found by qRT-PCR 2 or 24 hours after the memory recall test (p > 0.05 for all analyzed miRNAs, unpaired two-tailed Student’s *t* test) (Tables 4 and 5).

**Table 3:** Expression of the miR-132/-212 family 24 h after the memory test in the BLA of learned safety (LS) compared to learned fear (LF) mice.

miRNA	Mean LF ± SEM	Mean LS relative to LF ± SEM	p-value	t-value
miR-132	1 ± 0,05	1,06 ± 0,03	0,418	−0,837
miR-132*	1 ± 0,05	0,97 ± 0,02	0,558	0,599
miR-212-5p	1 ± 0,03	0,92 ± 0,03	0,125	1,634

Results were normalized to SNORD61 as reference RNA and plotted relative to the mean of controls (LF). Data are displayed as mean ± SEM. n = 7-8 per group.

**Table 4:** Expression of the miR-132/-212 family 2 h after the memory test in the hippocampus (HC) of learned safety (LS) compared to learned fear (LF) mice.

miRNA	Mean LF ± SEM	Mean LS relative to LF ± SEM	p-values	t-values
miR-132	1 ± 0,11	1,14 ± 0,16	0,514	−0,674
miR-132*	1 ± 0,04	0,99 ± 0,14	0,952	0,061
miR-212-5p	1 ± 0,11	1,09 ± 0,10	0,427	−0,824

Results were normalized to SNORD61 as reference RNA and plotted relative to the mean of controls (LF). Data are displayed as mean ± SEM. n = 7-8 per group.

**Table 5:** Expression of the miR-132/-212 family 24 h after the memory test in the hippocampus (HC) of learned safety (LS) compared to learned fear (LF) mice.

miRNA	Mean LF ± SEM	Mean LS relative to LF ± SEM	p-value	t-value
miR-132	1 ± 0,05	1,07 ± 0,02	0,214	−1.311
miR-132*	1 ± 0,05	1,00 ± 0,03	0,957	−0,055
miR-212-5p	1 ± 0,05	1,09 ± 0,03	0,346	−0,977

Results were normalized to SNORD61 as reference RNA and plotted relative to the mean of controls (LF). Data are displayed as mean ± SEM. n = 7–8 per group.

### The role of BLA miR-132 in learned safety

Considering strong indications from the literature for a pivotal role of the miR-212/-132 family in brain function (see for review^18^), we selected the most prominent representative, miR-132, for further experimentally assessment of its role in the behavioral expression of learned safety and related BLA neural activity. miR-132 (or miR-132-3p) is more commonly expressed than its “minor” sequence miR-132* (or miR-132-5p), which arises from the same pre-miR structure, and miR-212-5p, the 5p-arm of miR-212, and was therefore selected for all subsequent *in-vivo*, *ex-vivo* and *in-vitro* analyses.

Gain-of function experiments were conducted employing a miR-132 mimic, consisting of a double-stranded RNA oligonucleotide, which imitates the function of the endogenous, mature miRNA and is designed to favorably program the RISC complex with the active microRNA strand.

**Figure 3:**
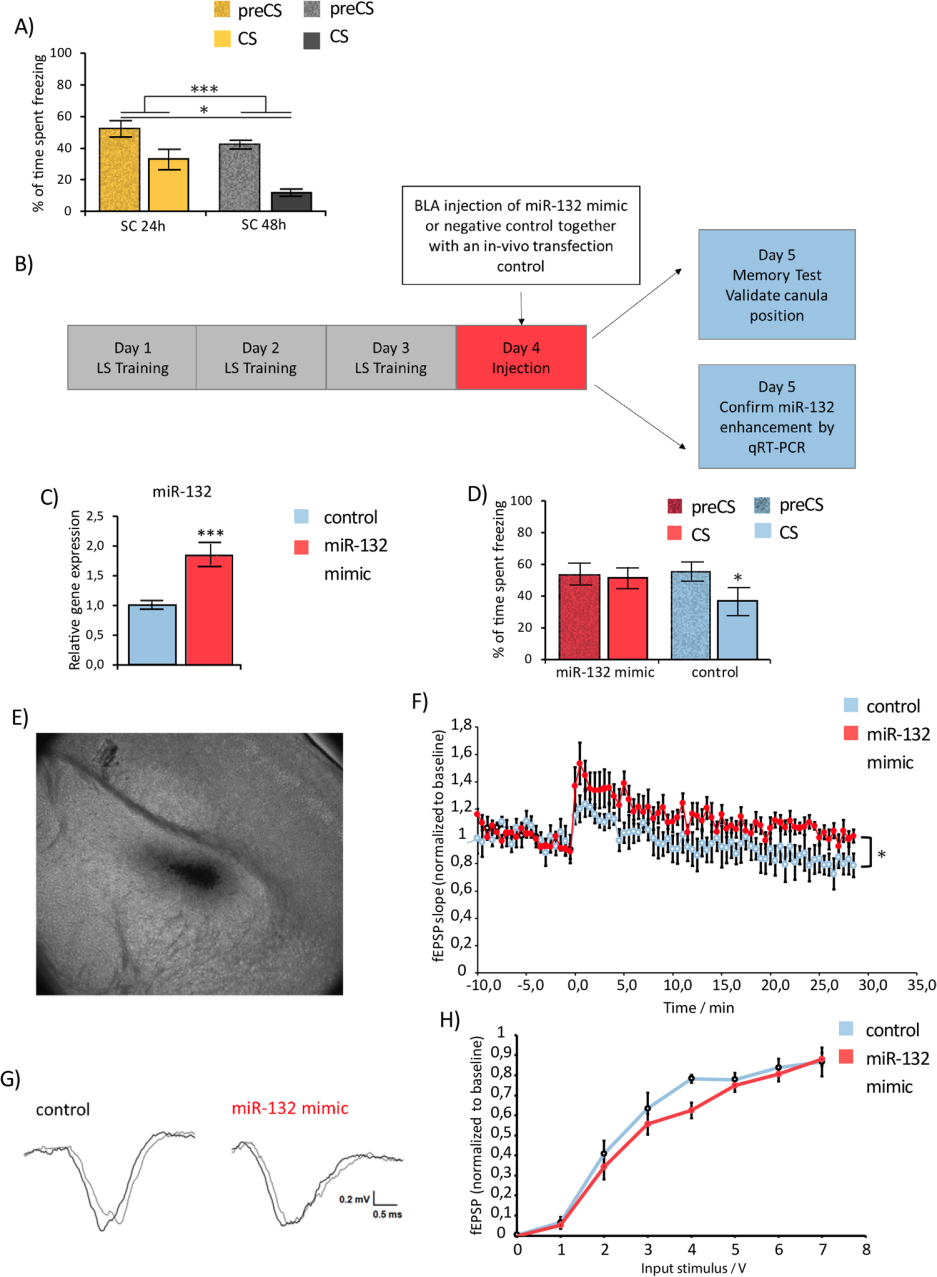
Effects of miR-132 enhancement on the behavioral expression of learned safety (LS) and on BLA long-term potentiation (LTP). (**A**) Percentage of time spent freezing before (preCS) and during (CS) presentation of the conditioned stimulus in LS trained mice 24 and 48 hours after the last training session (n = 9–13 per group). (**B**) Workflow for the experimental analysis for the role of miR-132 in learned safety (LS). Repeated measure ANOVA revealed a significant main effect of time points 24 h/48 h and a significant main effect of period preCS/CS. (**C**) miRNA expression 24 hours after the injection of a miRNA-132 mimic into the BLA. Relative expression of miR-132 determined by qRT-PCR in BLA tissue 24 hours after the injection of a miRNA-132 mimic. Results were normalized to SNORD61 as reference RNA and plotted relative to the mean of controls (injected with C. elegans miRNA mimic) (n = 10–13 per group). (**D**) Percentage of time spent freezing before (preCS) and during (CS) presentation of the conditioned stimulus in miR-132 mimic treated mice compared to controls (injected with C. elegans miRNA mimic). Mixed-model design ANOVA analysis revealed a significant interaction between time (preCS/CS) and injection (miR-132 mimic/control) (n = 12–14 per group). Data are displayed as mean ± SEM. *p < 0.05. (**E**) Toluol Blue injections via the implanted cannula targeting the BLA. Implantation of the cannula at the coordinates Anterior/Posterior −1.4 mm, Medial/Lateral +/− 3.15 mm, Dorsal/Ventral – 5.0 mm and subsequent injection of Toluol Blue via the cannula. (**F**) Effect of miR-132 enhancement in the BLA on long-term potentiation (LTP). Temporal course of the field excitatory postsynaptic potentials (fEPSP) normalized to baseline in BLA slices (n = 7–9 per group). Data are displayed as mean ± SEM. *p < 0.05 for time x treatment interaction. (**G**) Representative traces of field excitatory postsynaptic potentials (fEPSP) in miR-132 mimic treated versus control mice. (**H**) Analysis of basal synaptic transmission (input/output curves) recorded from amygdalar synapses in miR-132 mimic and control slices (n = 7–9 per group). All data are displayed as mean ± SEM. *p < 0.05, **p < 0.01, ***p < 0.001.

Intra-BLA delivery of the miR-132 mimic was used for the *in-vivo* transfection approach in order to examine the behavioral consequences of augmented BLA miR-132 levels. These experiments were conducted 24 hours after the last training day, as to prevent interference of the stress of the manipulation required to introduce the miRNA mimic into cannulated mice, on memory consolidation^28^. The memory recall test was scheduled 24 hours after the *in-vivo* transfections. To ensure that the memory for learned safety persisted over time and was properly expressed after 48 hours a memory recall test was carried out 24 and 48 hours after the last training session. Memory for learned safety, as indicated by significantly reduced freezing behavior to the conditioning context during the presence of the safety CS was present at both time-points (main effect of time points 24 h/48 h F(1, 20) = 6.05, p = 0.023; main effect of period preCS/CS F(1, 20) = 64.996, p = 0.0001; Fig. 3A).

Having confirmed the required experimental premises, the miR-132 mimic or negative control mimic (*C*.*elegans* miR-67) together with an *in-vivo* transfection reagent was injected into the BLA of cannulated mice 24 hours after the last training session (Fig. 3B). The *C*.*elegans* miR-67 mimic was selected as suitable negative control due to its minimal sequence identity with mouse miRNAs and the confirmed absence of detectable effects on examined mouse miRNA functions (www.http://dharmacon.gelifesciences.com/).

A significant enhancement in miR-132 levels in mimic as opposed to control animals was confirmed 24 hours after injection (t(21) = −5.081, p = 0.000; unpaired two-tailed Student’s *t* test; Fig. 3C). In a parallel group of animals we examined the behavioral consequences of increased BLA miR-132 and found that enhanced BLA miR-132 was associated with an impairment in the behavioral recall of learned safety in the memory test (F(1, 23) = 4.226, p = 0.037; mixed-model repeated-measure ANOVA: time (preCS/CS) x treatment (miR-132 mimic/*C*.*elegans* miR-67 control; Fig. 3D). Corrected localization of the cannula was confirmed via toluol blue staining and only animals with proper cannula placement were considered for the analysis (Fig. 3E).

The same approach was used to analyze the consequences of enhanced miR-132 levels on neural activity patterns in the BLA, previously associated with learned safety, as the recall of the safety CS has been found to induce long-lasting depression of activity in the lateral nucleus of the amygdala^3^. Analysis of long-term potentiation induced by stimulation of cortical inputs in the BLA 24 hours after injection of the miR-132 and control *C*. *elegans* miRNA mimic revealed a significantly augmented stability of the induced long-term potentiation over time in amygdala slices after *in-vivo* miR-132 enhancement (F(82, 984) = 1.371, p = 0.019, mixed-model repeated-measure ANOVA: time x treatment, miR-132 mimic/*C*.*elegans* miR-67 control (Fig. 3F,G)). No differences in the input-output relationship of evoked fEPSPs was detected between groups (p = 0.07, F(1, 13) = 4.03, n = 6–8 mice; Fig. 3H).

**Figure 4:**
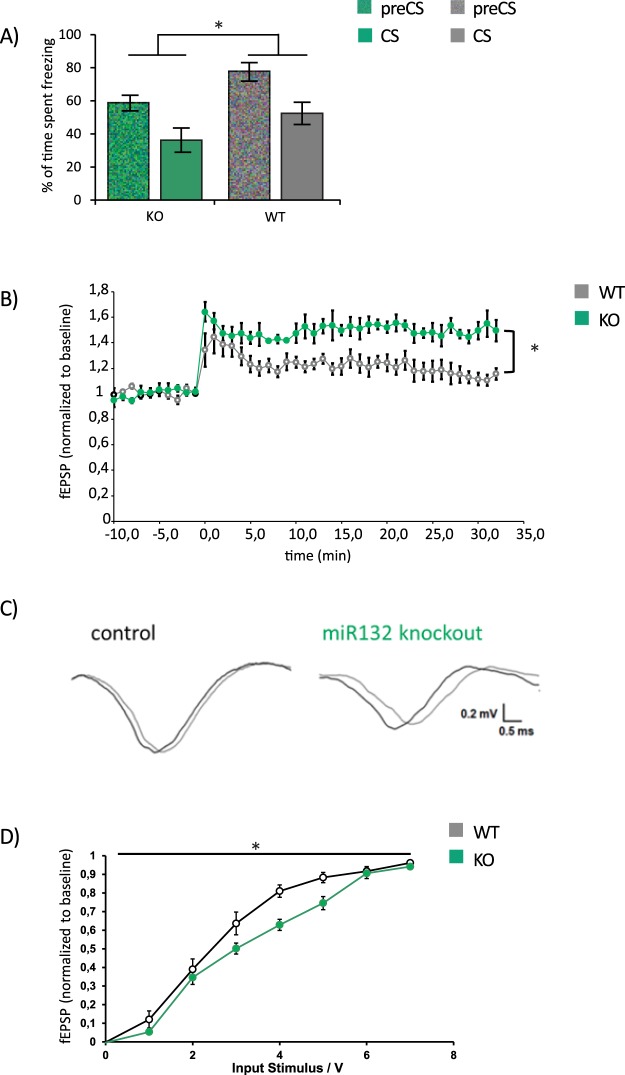
Behavioral expression of learned safety (LS) and BLA long-term potentiation (LTP) in miR-132 knockout mice. (**A**) Percentage of time spent freezing before (preCS) and during (CS) presentation of the conditioned stimulus in learned safety (LS) trained miR-132 knockout (KO) mice compared to wildtype littermate controls (WT). Mixed model design ANOVA revealed a significant effect of genotype on the percentage of time spent freezing in the memory trial (n = 10–9 per group). (**B**) Long term potentiation (LTP) in BLA slices of miR-132-KO compared to WT littermate controls (n = 4–6 per group). Temporal course of the field excitatory postsynaptic potentials (fEPSP) normalized to baseline is displayed. (**C**) Representative traces of field excitatory post synaptic potentials (fEPSP) in miR-132 KO and WT mice. (**D**) Analysis of basal synaptic transmission (input/output curves) recorded from amygdalar synapses in slices of miR-132 KO and WT mice (n = 4–6 per group). All data are displayed as mean ± SEM. *p < 0.05.

We next set out to complement gain-of-function experiments by the investigation of the role of the endogenous miRNA for learned safety *in-vivo* in a loss-of function approach. To this end we resorted to previously described miR-132/212 knockout mice (miR-132 KO)^19^. miR-132 KO and wildtype littermate controls (WT) were subjected to the learned safety paradigm and the performance in the recall test was evaluated. Mixed model repeated-measure ANOVA revealed a significant effect of genotype on the percentage of time spent freezing in the memory trial (F(1, 17) = 5.1656, p = 0.036; Fig. 4A). To mirror the gain-of-function set-up we also examined amygdala LTP *ex-vivo* in slices of miR-132/212 KO and WT mice. A significant main effect of genotype and a significant time x treatment interaction on amygdala long-term potentiation were observed in a mixed model repeated-measure ANOVA (main effect of genotype F(1, 8) = 12.2096, p = 0.0081; time x treatment interaction F(43, 344) = 3.911, p = 0.00001; Fig. 4B,C). An effect of genotype on the input-output relationship of evoked fEPSPs was observed (p = 0.03, F(1, 9) = 7.92) which was more evident at higher voltages than at input voltages used for LTP experiments (corresponding to ~30% of maximal input/output value) (Fig. 4D).

### miR-132 target gene analysis

Having confirmed an involvement for miR-132 for the behavioral display and the BLA neural signature of learned safety, we next went on to search for possibly relevant miR-132 target genes in this context. To this end, a dedicated bioinformatics workflow was designed to specifically increase the likelihood of identifying miR-132 target genes related to learned safety. The first inclusion benchmark for potential target genes comprised the prediction by more than one of the three used publically available miRNA target gene analysis tools (targetscan, MiRanda, EimMo3). Additional criteria for potential target genes were, if the predicted target gene belonged to a gene ontology considered of relevance for learned safety (eg. “multicellular response to stress”, “learning or memory”, neurotransmitter transport”, “regulation of neurotransmitter levels”). The next step consisted in determining whether the presumed target gene is expressed in the BLA, as determined by comparison to expressional data provided by the Allen brain atlas (http://www.brain-map.org). Finally, accessibility of the predicted miRNA target site on the mRNA for the gene silencing complex RISC was evaluated by structural analysis using the RNAfold WebServer (http://rna.tbi.univie.ac.at/cgi-bin/RNAWebSuite/RNAfold.cgi)^29^. This analysis is based on the observation that miRNA binding sites are located exclusively in “unstable”, linear mRNA structures. Due to their linearity resulting from less internal folding of the mRNA, these regions possess higher accessibility for the RISC complex^30,31^. The RNAfold server enables prediction of internal folding of the miRNA target site on the basis of its nucleotide sequence. Additionally, the RNAfold server predicts minimum free energy (dG) values of a given nucleotide sequence, thereby providing a numeric value representing the degree of internal mRNA folding. A high likelihood miRNA target site contains a dG value higher than the average dG value of 60 random 3′ UTRs (70 bp each) in the corresponding species, which for the mouse is −13.4 kcal/mol^29^.

For miR-132 three target genes containing miRNA target sites with dG value higher than −13.4 kcal/mol, namely GABA transporter 1 (GAT1), MeCP2 (Methyl-CpG-Binding Protein 2) and Phosphatase and Tensin homolog (PTEN) were predicted. PTEN carries three miRNA target sites with dG values higher than −13.4 kcal/mol while MeCP2 - an already validated target gene for miR-132 and miR-212^18^ and GAT1 both contain one such miRNA target site (Table 6).

**Table 6:** High likelihood targets for miR-132 determined by minimum free energy (dG) values representing structural accessibility of miR-132 binding sites on the target gene.

miRNA	mRNA	binding site	5′ 70 bp dG	3′ 70 bp dG
miR-132	GAT1 (Slc6a1)	I	−10,2	−23,7
		II	−19,4	−14,8
		III	−16	−23
miR-132	MeCP2	I	−10,3	−19,1
miR-132	PTEN	I	−5,2	−7,7
		II	−12,7	−9,6
		III	−12,6	−12,6

High likelihood target genes for miR-132 with binding sites containing dG values higher than the average dG value of 60 random 3′ UTRs (70 bp each) in the corresponding species, which for the mouse is −13.4 kcal/mol.

**Figure 5:**
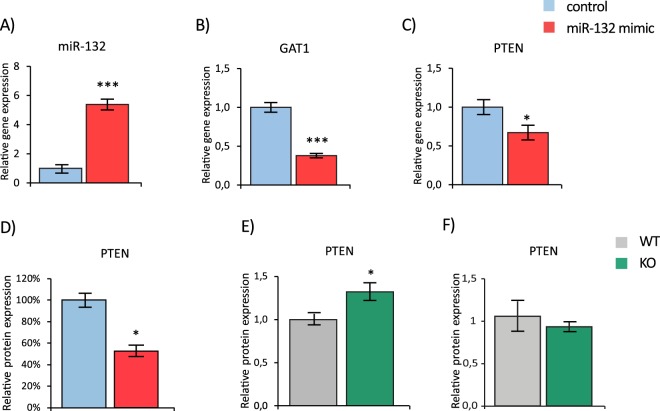
miR-132 target gene analysis in response to miRNA mimic transfection and in miR-132 KO mice. (**A**) Relative miRNA expression of miR-132 in 3t3 cells 24 hours after transfection with a miR-132 mimic. Results were analyzed by qRT-PCR and were normalized to the reference gene SNORD 61. Relative mRNA expression of (**B**) GAT1 and (**C**) PTEN in 3t3 cells 24 hours after transfection with a miR-132 mimic (n = 6 per group). Results were analyzed by qRT-PCR and were normalized to the reference gene beta-actin. (**D**) Relative protein expression of PTEN in 3t3 cells 24 hours after transfection with a miR-132 mimic. (n = 2–3 per group) determined by Western Blot. Data are displayed as mean ± SEM. *p < 0.05. (**E**,**F**) Relative protein expression of PTEN in BLA tissue of miR-132 KO mice compared to WT control und (**E**) baseline conditions and (**F**) in learned safety trained mice (n = 3–4 per group) determined by Western Blot. All data are displayed as mean ± SEM. *p < 0.05, ***p < 0.001.

**Figure 6:**
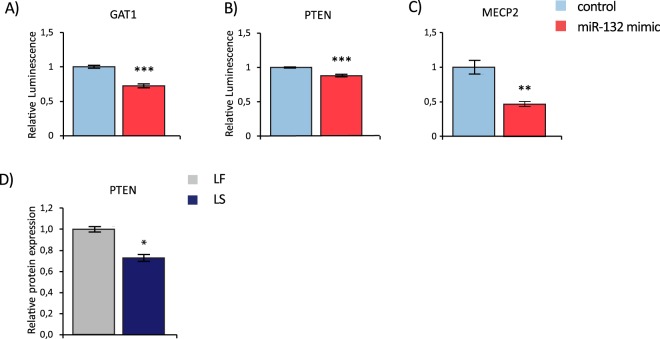
miR-132 target gene expression in learned safety. Relative luminescence in the renilla luciferase assay comparing the effects of miR-132 mimic versus control (C. elegans miRNA mimic) on the 3′UTR region of putative miR-132 target genes fused to the renilla luciferase gene: (**A**) GAT1 (**B**) PTEN and (**C**) MeCP2 (control) (n = 4 per group). (**D**) Relative protein expression of PTEN in BLA tissue of learned safety (LS) trained mice compared to control (learned fear (LF) trained mice) (n = 4 per group) as analyzed by Western Blot. All data are displayed as mean ± SEM. *p < 0.05, **p < 0.01, ***p < 0.001.

We therefore went on to experimentally validate the selected target genes in an *in-vitro* model, using the murine 3t3 cell line, endogenously expressing both GAT1 and PTEN^32,33^. Significantly and more than 5-fold increased miR-132 levels were observed 24 hours after transfection of the miR-132 mimic as compared to the control (*C*.*elegans* miR-67) (t(10) = −19.266, p = 0.000; unpaired two-tailed Student’s *t* test (Fig. 5A)). In parallel, the expression of both GAT1 and PTEN mRNA levels was significantly reduced in the mimic as compared to the control transfected cells, suggesting both genes as targets of miR-132 (GAT1: t(10) = 9.036, p = 0.000; PTEN: t(10) = 2.444, p = 0.035; unpaired two-tailed Student’s *t* test (Fig. 5B,C)). We exemplarily verified the reduction of PTEN levels after transfection with the mimic at the protein level (PTEN: t(3) = 4.066, p = 0.027; Fig. 5D). Reversely, significantly higher PTEN protein levels were observed in miR-132 KO than in WT control mice under baseline conditions (PTEN: t(5) = −2.589, p = 0.049; Fig. 5E) while no differences were detected between genotypes after safety training (PTEN: t(8) = 0.676, p = 0.539; unpaired two-tailed Student’s *t* test (Fig. 5F)). Finally, we used a luciferase reporter assay in order to confirm the direct regulatory effect of miR-132 on levels of GAT1, PTEN and MeCP2 as positive control. The co-expression of the miR-132 mimic, but not the control (*C*.*elegans* miR-67) and a plasmid with the 3′ untranslated region of either GAT1, PTEN or MeCP2 fused to the firefly luciferase gene significantly reduced luminescence representing Renilla luciferase activity (GAT1: t(6) = 3.875, p = 0.008; PTEN: t(6) = 6.247, p = 0.001; MeCP2: t(6) = 5.081, p = 0.002; unpaired two-tailed Student’s *t* test; Fig. 6A–C).

In order to further characterize the role of the identified targets in the learned safety behavior we examined the expression of PTEN in amygdala tissue of learned safety and learned fear trained animals 12 hours after the memory recall test. Western Blot analysis provided evidence for a reduction in PTEN protein levels in learned safety trained mice (t(6) = −3,284, p = 0.017; Fig. 6D). These data suggest a complex involvement and intricate molecular regulation of miR-132 and its effector genes in the neural mechanisms of learned safety.

## Discussion

Our understanding of the neurobiological mechanisms underlying the most complex and excruciating mental illnesses, including depression, remains incomplete, hampering the development of novel diagnostic and therapeutic approaches. Spearheaded by the RDoC project of the NIMH, the scientific focus has shifted recently towards approaches aiming to dissect entire psychopathological entities, such as depression, into different dimensions of basic brain functioning underlying behavior. Along these lines, individual intermediate phenotypes may be established which are more amenable to experimental in-depth characterization at various levels, ranging from the analysis of neural circuitries to the investigation of cellular and molecular principles. The RDoC matrix explicitly lists “fear” and “threat” as relevant constructs within the “negative valence” system and internal or external stimuli predicting the possibility of the experience of reward stimuli as applicable sub-construct within the “positive valence” system (https://www.nimh.nih.gov/research-priorities/rdoc/constructs/index.shtml). As such, learned safety, a fear inhibitory paradigm with defined rewarding and antidepressant-like effects^2,3^ can be integrated into the positive valence system and may represent a valuable tool for the investigation of a distinct behavioral function relevant for several psychiatric disorders.

The present study shows that learned safety is associated with a distinguished signature of 6 miRNAs in the mouse BLA. Interestingly, the expression of all safety-related miRNAs (miR-132, miR-132*, miR-212-5p, miR-100, miR-92a and miR-15b) was reduced in learned safety, suggesting the possibility for a common regulatory mechanism; eg. decreased levels of all six safety-related miRNAs may reflect a reduction in the activity of a shared controlling modulatory element, such as a transcription factor. Significant difference in the BLA levels of five of these miRNAs (miR-132, miR-132*, miR-212-5p, miR-100 and miR-15b) was revealed when comparing learned safety but not learned fear groups to their respective shock alone controls (i.e. matching training paradigm). It can be speculated that the higher number of shocks received in the learned safety-matched shock alone controls as compared to the learned fear matched shock alone controls may induce a higher level of arousal during the memory recall, rendering the re-exposure without further negative consequence a reinforcing experience^34^. Along these lines it has to be noted that for learned safety, as is the case for many other (behavioral) paradigms, the issue of determining the proper controls is critical and not trivial. We have, as in our previous study initially describing the antidepressant-like effects on learned safety^2^, opted to use a learned fear paradigm, in which the number of CS and US presentations have been matched to the learned safety protocol, as primary control. The rationale for this choice is based upon the equal number of shock and tone presentations in the two groups differing solely in the contingency between the two stimuli and was therefore considered to constitute the most appropriate control for the present set of experiments.

Interestingly, the regulation in the expression of the identified miRNAs by the memory recall of learned safety resulted in a rapid (2 hours), but not sustained (24 hours) reduction in the BLA of trained animals. This very quick decrease may be caused by several mechanisms including stimulus-induced fast decay of mature neuronal miRNA as previously demonstrated^35,36^. Of note, the observed enhancement of BLA CREB levels in learned safety trained mice suggests the possibility of a rather complex interrelationship between miR132 and CREB in learned safety. While miR-132 is induced by CREB it also forms part of a negative feedback mechanisms to restrain CREB-dependent transcription^37^ including regulation of its own regulation of its own expressional levels^38^.

The finding that expressional changes of safety-related miRNAs were found only in the BLA, but not in the hippocampus, supports the notion that the BLA and the hippocampus serve distinct functions in the neural circuit of learned safety^4^. As such, the involvement of the miR-212/-132 family in learned safety may more directly relate to the positive valence of the learned safety signal, which, together with its rewarding properties, seems to be distinctively regulated by a determined set of amygdala neurons^9^ and less to the inhibitory gating mechanisms attributed to the hippocampus^39^. However, it needs to be taken into consideration that even within the BLA several microcircuits exist^9,40–43^ which, may be differentially involved in learned safety and contribute distinct gene expression profiles which were masked in the present approach by considering the BLA as whole.

The pivotal role of miR-132 in brain function led us to further investigate the involvement of this miRNA in the behavioral and neural expression of learned safety. Indeed, miR-132 likely constitutes one of the most intensively studied miRNAs in the context of synaptic activity, synaptic plasticity and learning and memory (see for review^44^).

Based upon the reduction of BLA miR-132 expression in learned safety, we hypothesized that enhancing BLA miR-132 ought to constitute an inhibitory constraint on the performance in the recall test and confirmed this hypothesis in mice after intra-BLA injection of a miR-132 mimic. Reversely, in miR-132 KO we determined significantly lower levels of freezing during the memory recall test. Interestingly, behavioral observations in different genetic mouse models of altered miR-132 expression have yielded, in part surprising, insights into the role of miR-132 in learning and memory. The here employed conventional, full-body knock out of the miR-132/212 locus has led to the generation of mice with impaired recognition and spatial memory^45^. Curiously, forebrain-specific overexpression of miR-132 also led to aberrant learning and memory performance in mice^46^. While it may at first be considered confusing that gain-of-function and loss-of-function approaches have comparable outcomes, it has been concluded that this observation is reflective of the biological principle that the levels of products miR-132/212 locus must remain within a controlled range in order to ensure physiological neuronal functioning^44^.

We then followed up on the behavioral demonstrations by investigating a role of BLA miR-132 as restrictive constraint at the neural network level and observed that local increases in miR-132 strengthened BLA LTP induced by stimulation of cortical afferents. Interestingly, the constitutive deletion of miR-132 in KO mice also resulted in an augmentation of theta-burst induced BLA LTP, paralleling a previous report in hippocampal slices in this mouse line^19^. This observation is intriguing and whilst the precise operative mechanisms of miR-132 in BLA LTP cannot be delineated from the present set of experiments it can be speculated that the reduction of miR-132 in trained animals may serve to augment the potential of the safety CS to repress neural plasticity in the BLA^1^. Indeed, miR-132 has been reported to constitute a major regulator of basal synaptic activity and functional plasticity (see for review^44,47^), in part through the regulation of neuronal morphology and growth^48–50^. This critical role of miR-132 for synaptic function might also explain the small alterations in basal synaptic transmission in amygdala slices of miR-312 KO mice specifically at high voltage inputs which is in agreement with previous observations in hippocampal slices^51^. However, since compared to WT controls miR-312 KO slices displayed lower responses in the input-output curve, but larger increases in the slope of fEPSPs in the LTP experiments, results from the input-output analysis are unlikely to present a confound for the interpretation of LTP experiments.

A thorough electrophysiological investigation at the single cell level elegantly demonstrated in freely moving rats, that the safety CS leads to the differential firing pattern in a defined set of neurons in the basal amygdala, in comparison to fear and overlapping with reward stimuli^9^. As both increases and decreases in the firing rate of defined neuronal population were observed, future studies may be designed in order to specifically address a potential overlap in the expressional pattern of miR-132 with neurons displaying reduced activity in response to the safety CS and their corresponding molecular identity with regards to GABAergic and glutamatergic neurotransmission.

In order to shed light onto the molecular mechanisms by which miR-132 may be involved in the regulation of BLA function and the expression of learned safety, we turned towards investigating potential miR-132 target genes. Using a multilevel, integrated coherent bioinformatics workflow led to the nomination of three high likelihood target genes for miR-132 with possible relevance for learned safety: the already experimentally validated target gene MeCP2^52^ and the predicted, potential target genes GAT1 and PTEN. We used two independent, selective *in-vitro* approaches in order to demonstrate the regulation of GAT1 and PTEN by miR-132; indeed, both miR-132 mimic and luciferase assays experimentally confirm GAT1 and PTEN as miR-132 target genes. The expression of PTEN was reduced in the BLA tissue of learned safety trained mice after the memory recall test which parallels a similar observation in the learned safety-related fear extinction paradigm^53^. Together these data suggest that an intricate network of transcriptional and posttranscriptional mechanisms involving several miRNAs (eg. also miR-144-3p^53^) and other regulatory principles may be in place to fine tune the expression of a high relevance neural target gene.

Hence, we postulate that, in the framework of the behavioral expression and neural signature of learned safety in the BLA, miR-132 may determine neural excitability and synaptic plasticity through modulation of the expression of its target genes, including GAT1 and PTEN, both of which have also been identified as molecular key players in fear extinction, a learned safety-related fear inhibitory paradigm^16,54^. Further experiments, including the temporary controlled inhibition of miR-132 *in-vivo* - complementing the constitutive deletion experiments in the miR-132 KO mice - as well as *in-vivo* knockdown approaches centering on GAT1 and PTEN are required in order to delineate in greater detail the molecular involvement of miR-132 and its target genes in learned safety in the future.

Above all, we were able to provide the first experimental assessment of the involvement of miRNA expression in learned safety, which we propose as relevant construct within the positive valence system of the RDoc matrix. Following the suggested in-depth characterization for individual intermediate phenotypes, we have carried out a comprehensive assessment from the behavioral systemic, to the neural network and molecular levels and propose a central role of miR-132 in the regulation of learned safety. The suggested function of miR-132 as inhibitory and signature molecule may be considered in future preclinical and translational approaches testing the transdiagnostic relevance of learned safety as intermediate phenotype across fear and stress-related disorders.
